# Supplementary material for: Epidemiology of Secondary Warm Autoimmune Haemolytic Anaemia—A Systematic Review and Meta-Analysis
Source: J Clin Med. 2021 Mar 17;10(6):1244. doi: 10.3390/jcm10061244 (PMC8002719; doi:10.3390/jcm10061244)
Supplement: Supplementary file 1 [file jcm-10-01244-s001.pdf]

Supplementary materials

Figure S1 – Graphic presentation of median age at diagnosis, age characteristics of the respective studies, and number of included patients in the studies

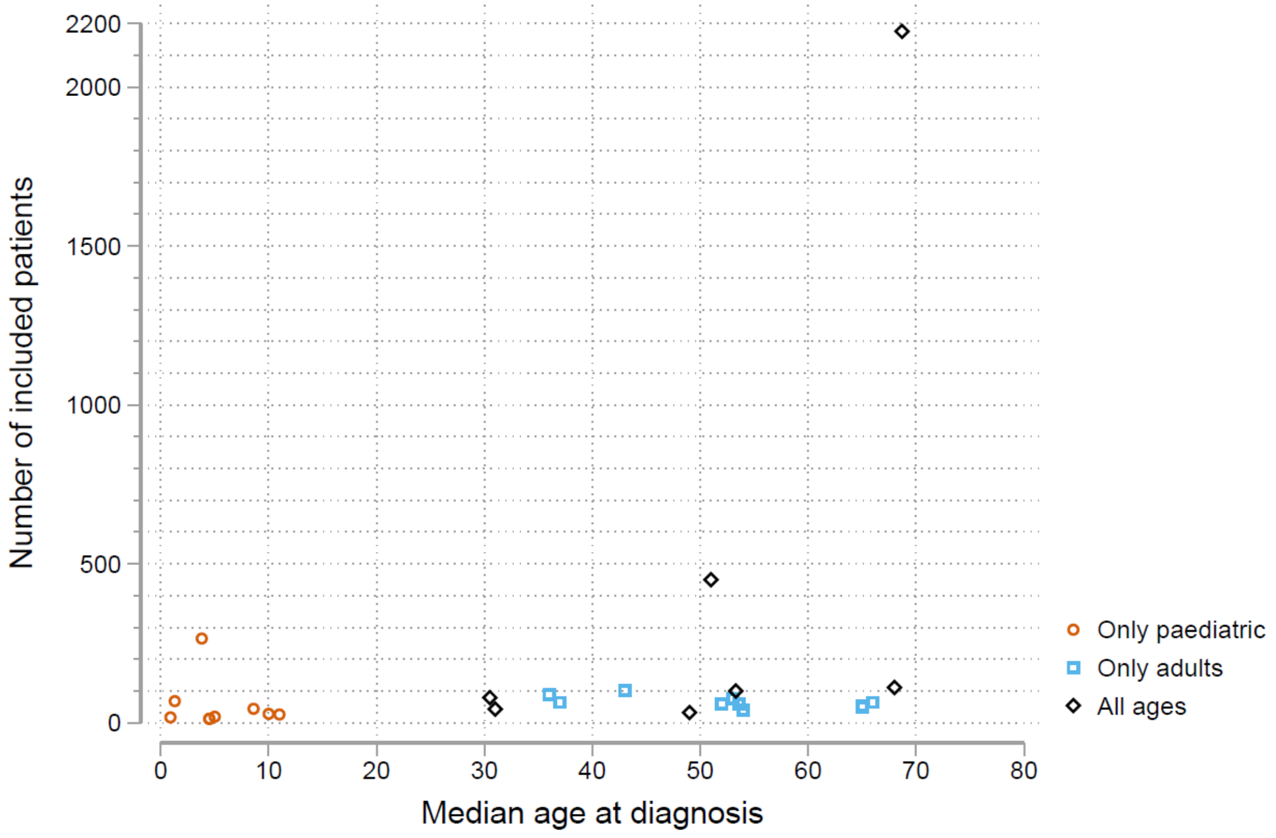

Figure S1: This graph shows the median age at diagnosis and the number of patients included in the studies. Note that one study includes far more patients than the remaining studies.[4] Orange circle: studies including only paediatric patients included, blue square: studies including only adult patients included, white diamond: studies including patients of all ages.

Table S1. Complete list of all mentioned associated diseases for secondary warm autoimmune haemolytic anaemia (wAIHA) and the total number of mentions.

**Associated disease**	**Number of reported patients**
Systemic lupus erythematosus	220
Evans syndrome	156
Connective tissue disease (mostly systemic lupus erythematosus)	93
Infection	63
Non-Hodgkins lymphomas	59
Lymphoid diseases	45
Chronic lymphoid leukaemia	38
Unspecified	36

Autoimmune diseases	32
B-cell lymphomas	32
Reumatoid arthritis	26
Antiphospholipid syndrome	21
Diabetes mellitus	20
End organ failure (renal)	19
Lymphoma	18
Sjøgrens disease	17
Autoimmune hepatitis	14
Haematological malignancies	14
Solid tumor	14
Anaemia (not otherwise specified)	12
Myelodysplastic syndrome	12
Over-lap syndrome	12
Plasma cell leukaemia	12
Hepatitis B virus infection	10
Cytomegalovirus infection	10
Humoral deficiency	10
Common variable immune deficiency	9
Human immunodeficiency virus (HIV) infection	9
Epstein Barr virus	8
Primary immunodeficiency disorder	8
Tuberculosis	8
Allogeneic haematopoietic stem cell transplantation	7
Colitis ulcerosa	7
Enteritis	7
Hodgkin lymphomas	7
Surgery	7
Autoimmune lymphoproliferative syndrome	6
Leukaemia (not otherwise specified)	6
Lymphoproliferative diseases	6
Hepatitis C virus infection	5
Renal cell carcinoma	5
Thyroid disease	5
ANCA associated vasculitis	4
Endocrine disease (not otherwise specified)	4
Myeloid haematological disease	4
Acute lymphoid leukaemia	3
Aplastic anaemia	3
Combined immunodeficiency	3
Hashimoto thyreoiditis	3
Nephritis	3
Primary immune thrombocytopenia (not registered as ES)	3
Acute granulocytic leukaemia	2
Chronic granulocytic leukaemia	2

Colon cancer	2
Giant cell hepatitis	2
Grave's disease	2
Hyperthyroidism	2
Hypogammaglobulemia	2
Isolated antinuclear antibodies	2
Liver cirrhosis	2
Myelofibrosis	2
Psoriatic arthritis	2
Rubella	2
Systemic scleroderma	2
Sickle cell disease	2
Visceral leishmaniasis infection	2
Vitelligo	2
Wiskott–Aldrich syndrome	2
Adenosine deaminase deficiency	1
Adult morbus Still	1
Ankylosing spondylitis	1
Autoimmune lymphoproliferative syndrome	1
Bare lymphocyte syndrome type II	1
Breast cancer	1
Carcinoma	1
Castleman disease	1
Cellulitis	1
Chronic granulocytic leukaemia	1
Chronic liver disease	1
Chronic lymphoproliferative disease (NK cell type)	1
Chronic myeloid leukaemia	1
Chronic myelogenous leukemia	1
Chronic myelomonocytic leukaemia	1
CNS tumor	1
Congenital dyserythropoietic anaemia	1
Crohn's disease	1
Dermatomyositis	1
DiGeorge syndrome	1
Dyskeratosis congenita	1
Epstein Barr virus associated T-lymphoproliferative disease	1
Gastric cancer	1
GINS1 mutation	1
Hairy cell leukaemia	1
Hemophagocytic syndrome	1
Haemorrhagic rectocolitis	1
Hepatic cancer	1
Hepatitis A virus	1
Hepatitis E virus infection	1

Herpes virus infection (not otherwise specified)	1
HLA II deficiency	1
Immune neutropaenia (not registered as Evans syndrome)	1
Langerhans cell histiocytosis	1
Lymphadenopathy	1
Mycosis fungoides	1
Mycoplasma infection	1
Non-tuberculous mycobacteria infection	1
Ovarian cancer	1
Polyglandular autoimmune disorder	1
Primary sclerosing cholangitis	1
Prostatic cancer	1
Rheumatic disease	1
Soft tissue infection	1
Status post orthotopic liver transplantation	1
Tongue cancer	1
X-linked lymphoproliferative disease	1

Table S1: Complete list of all 114 associated diseases mentioned in all included studies, from the most frequently mentioned to the least frequently mentioned. Some of the syndromes are overlapping, as some patients are mentioned to have more than one presumed associated disease.
